# Infiltrated thin film structure with hydrogel-mediated precursor ink for durable SOFCs

**DOI:** 10.1038/s41598-021-86572-w

**Published:** 2021-03-29

**Authors:** Sangyeon Hwang, Mingi Choi, Jongseo Lee, Giho Kang, Seo Ju Kim, Baekhoon Seong, Hyungdong Lee, Wonyoung Lee, Doyoung Byun

**Affiliations:** School of Mechanical Engineering, Sungkyunkwan University, 2066, Seobu-ro, Jangan-gu, Suwon-si, Gyeonggi-do 16419 Republic of Korea

**Keywords:** Fuel cells, Organic-inorganic nanostructures, Gels and hydrogels, Fuel cells

## Abstract

The hydrogel of biomolecule-assisted metal/organic complex has the superior ability to form a uniform, continuous, and densely integrated structure, which is necessary for fine thin film fabrication. As a representative of nature-originated polymers with abundant reactive side chains, we select the gelatin molecule as an element for weaving the metal cations. Here, we demonstrate the interaction between the metal cation and gelatin molecules, and associate it with coating quality. We investigate the rheological property of gelatin solutions interacting with metal cation from the view of cross-linking and denaturing of gelatin molecules. Also, we quantitatively compare the corresponding interactions by monitoring the absorbance spectrum of the cation. The coated porous structure is systematically investigated from the infiltration of gelatin-mediated Gd_0.2_Ce_0.8_O_2−δ_ (GDC) precursor into Sm_0.5_Sr_0.5_CoO_3−δ_ (SSC) porous scaffold. By applying the actively interacting gelatin–GDC system, we achieve a thin film of GDC on SSC with excellent uniformity. Compare to the discrete coating from the typical infiltration process, the optimized thin film coated structure shows enhanced performance and stability.

## Introduction

Biomolecules have received attention as functional materials for various applications, because of their sustainability and unique properties, in comparison with fossil fuel-derived materials. Since they have more variety in constituent elements than hydrocarbon molecules, various interactions between biomolecules and inorganics can be utilized to compose complex structure. The biomolecules can fulfill many parts of interactions in organic–inorganic complex systems, due to their abundant reactive side chains. To understand and utilize the organic–inorganic complexes, several investigations have been conducted regarding the interaction between biomolecules and metallic elements. From monomer to polymer, the metal complexation has been investigated for various applications^1–3^. Small molecules, such as amino acid and peptides, have been used for chelation and dispersion of metallic components^4, 5^. Meanwhile, the biomolecules with longer chain, such as chitosan, alginate, DNA, and gelatin, have more complicated structure, and the single polymer molecule can interact with numerous metal cations^6^. The ability to interact with other materials and their inherent self-assembling characteristics enable the versatile design of complex states (e.g., fluid, gel, and crystal)^7^. Furthermore, the designable metal/organic complex would be advantageous to synthesize further functional materials after calcination with the desired component morphology and distribution^8^.

Among such biopolymers, gelatin is a promising material, due to its superior compatibility and coordinating capability with inorganic materials. Easy processability with high solubility in polar solvents, adjustable chemical properties by simple reactions, and broad selectivity of physical properties, such as gel strength, viscosity, and melting temperature, allow extensive potential applications. In addition, the abundant sources of gelatin in nature allow diversification of raw materials, unlike synthetic polymers. Due to its superior properties, gelatin has been employed as a base material for bio-applications and binding material for the synthesis of inorganic compounds. Seong et al. reported a dense 2-dimensional metal structure by applying self-layering and crystallization of metal-ion/gelatin complex^9^. In addition, Wang et al. demonstrated a homogeneous polymer/metal complex system by applying calcined gelatin-based iron precursor solution to control the metal oxide/carbon hybrid structure^10^. Unlike the particle size of iron oxide (~ 200 nm) from a typical synthesis, the particle size of gelatin-mediated synthesis was several nanometers^10^. However, the pure metal oxide nano-structures without organic or carbon residues that were started from a gelatin-mediated complex system have never been reported. Nanostructured oxides, such as thin film, are becoming important in diverse fields of engineering from the view of diversification and reinforcement of the interfacial configuration^11–13^. The oxide film fabrication basically requires high calcination temperature above 500 °C. Meanwhile, the high-temperature energy conversion devices, such as solid oxide fuel cells (SOFCs), are the representative field that demands thin oxide film. Conventional vacuum deposition techniques, such as atomic layer deposition (ALD), have been used to cover the surface of reactive and unstable electrodes. Thin ZrO_2_ coating (~ 10 nm) was reported to be able to suppress the degradation of the La_0.6_Sr_0.4_CoO_3‑δ_ electrode surface and enhance the durability for long-term operation^14^. The impressive results allow new insights into the development of highly durable SOFCs with well-structured coating layer in a thickness of only a few nanometers. However, the implementing technique for the porous cathodes with vacuum deposition tool hinders general application, because of the long deposition time, and limitation of the available fabrication area. Hence, an effective thin film coating technique without vacuum apparatus is required for the commoditization of durable cells. Although there have been attempts to achieve fine coating using infiltration process, continuous film could be achieved by repeated infiltration and controlled solvent drying rate, which are still not appropriate for mass production^15–18^.

In this paper, we demonstrate a wet chemical coating for preparing a thin film of metal oxide on a microporous structure by employing compact and homogeneous distribution of organic–inorganic complex, which is attributed to gelatin–cation interaction. For the investigation, SSC scaffold and GDC precursor were selected as the cathode material and infiltrated material, since the combination of SSC and doped ceria is known to exhibit improved persistency, with maintaining the microscopic structure. The continuous nanoscale film was achieved by applying the sol–gel method of homogeneous gelatin/metal complex. Glycine-based GDC precursor solution was also investigated for comparison with monomer-based coating. The interaction between organic molecules and cations was monitored and compared by measurement of the viscosity and absorbance of each solution. Both mechanical and optical properties were significantly affected by the ratio between gelatin and GDC precursor, while the corresponding mixtures of glycine and GDC precursor had slight differences. The higher degree of interaction in the gelatin-based solution was attributed to the fine coating of GDC on the SSC scaffold. The electrochemical performance of initial and long-term (100 h) operation was conducted for different film morphology, to evaluate the effect of the structural modification. The fine GDC coating achieved by gelatin-based solution infiltration showed a reduced degradation rate of the electrode. We expect that our results can provide a sustainable and effective solution-based coating method for the universal synthesis of nanostructured metallic compounds.

## Experimental details

### Infiltration solution preparation

Gelatin type A (Sigma-Aldrich, for microbiology) was dissolved in water/ethanol (60/40, v/v) at 80 °C with stirring. After cooling the gelatin solution to room temperature (R.T.), gadolinium nitrate hexahydrate (Gd(NO_3_)_3_∙6H_2_O) and cerium nitrate hexahydrate (Ce(NO_3_)_3_∙6H_2_O) were added to the solution at a molar rate of 1:9, while the molar concentration of cation was (0.025–0.4) M. The pre-cooled gelatin solution had no flowability, since gelation of the triple helix structures of secondary phase molecules occurred. However, after adding metal precursors, ions interrupted the crosslinking of gelatin molecules, and hence the secondary phases were denatured to become a sol. Monomer-based solution was prepared with only replacing gelatin by glycine (Sigma-Aldrich). Since the quantities of reactive groups of gelatin, such as amino, carboxyl, and hydroxyl groups, are about ~ 3 mol per 1 g of gelatin, ~ 6 times more gelatin by mass was needed to match the quantity of reactive groups of glycine (MW = 75.06)^19^. In a common glycine–nitrate process, the mole ratio between the nitrates and glycine of 1:2 for trivalent metal ions is proposed, which corresponds to ~ 0.5 wt% of glycine for 0.05 M of GDC precursor solution^20^.

### Infiltration process

The precursor solutions were infused into the SSC porous scaffold of ~ 200 nm particle size and 8 μm thickness sintered on a GDC pellet. The 2.5 μL of GDC precursor solution was gently dropped onto the electrode by micropipette. It is important not to leave residues on top of the layer for gas permeability and current collection. The solution completely permeated through the layer within 5 min, without excessive solution remaining on the electrode surface. The cell was placed under vacuum desiccator for 15 min, to get rid of vacancies inside the electrode. Then, the cell was dried for 30 min in air at 200 °C with a fast heating rate of 350 °C/h, to evaporate all the solvents. Subsequently, the heating temperature was slowly increased to 800 °C with a heating rate of 100 °C/h, and maintained for 3 h. After cooling the cell by natural convection, the thin coating on the porous electrode was achieved.

### Characterization methods

Microscopic morphology of the coated electrode was examined by field effect secondary electron microscopy (FESEM, JEOL), according to the different solutions and thermal degradation. The viscosity of solutions with different concentration of gelatin and ions was measured by rheometer (TA Instruments). The morphology and crystalline phase of GDC film were characterized by transmission electron microscopy (HRTEM, JEOL). The interaction between gelatin molecule and metal ions was investigated by Raman spectrometry (Horiba Jobin Yvon, LabRam Aramis) using 785 nm diode laser. The absorbance of solutions with different concentration of gelatin was measured by UV–VIS-NIR spectrometry (Agilent). Electrochemical impedance spectroscopy (EIS) measurements for the symmetric cells were performed using a custom-made test station. X-ray photoelectron spectroscopy (XPS, ESCALab 250 XPS spectrometer, VG Scientific Instruments) with a monochromatic Al Kα source and Auger electron spectroscopy (AES, ULVAC-PHI, PHI 700) were used to characterize the Sr 3d photoelectron spectra and Sr *LMM* auger emission, respectively.

## Result and discussions

**Figure 1:**
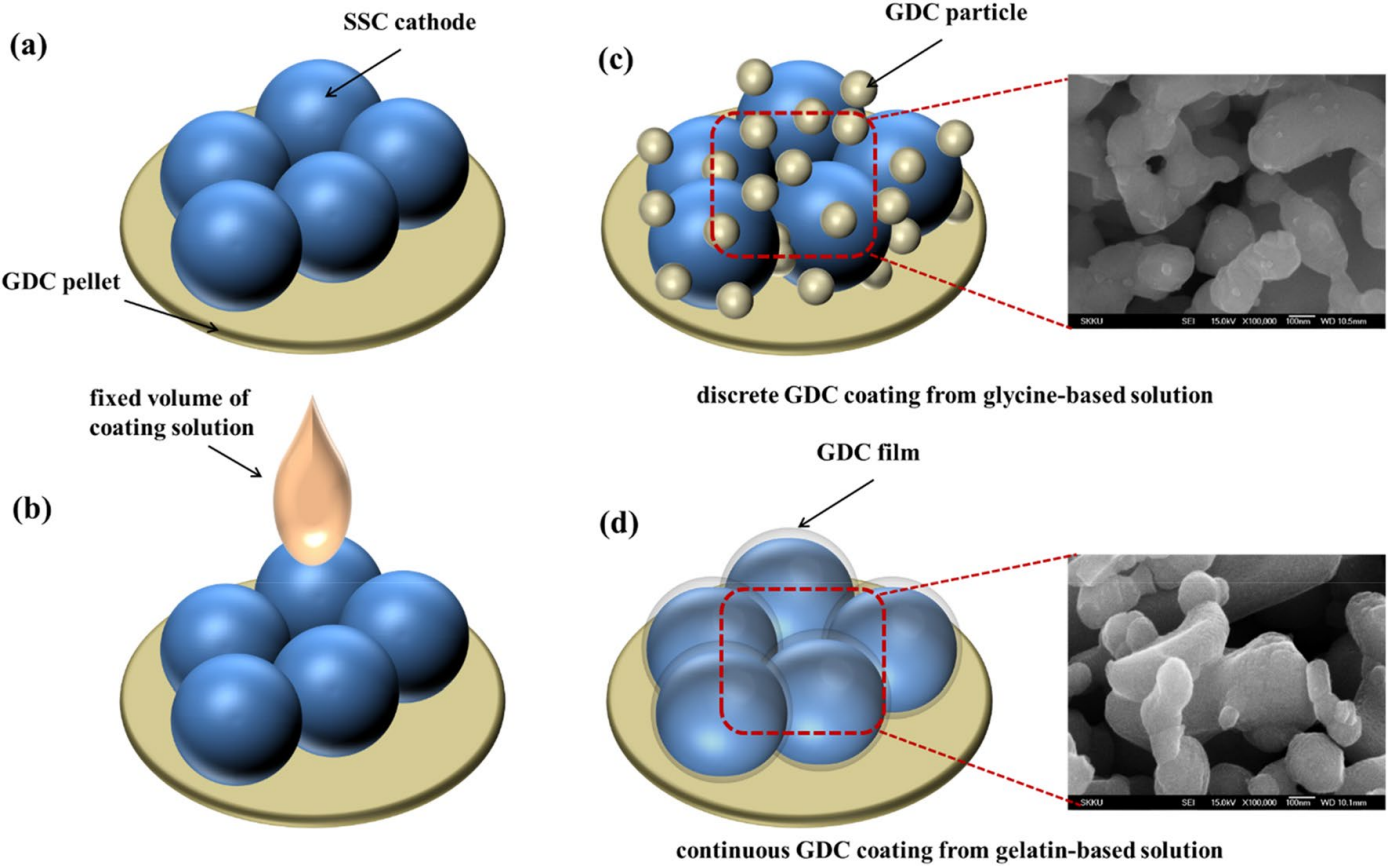
Overall process of surface coating onto the SSC cathode using infiltration process and the resultant SEM images of coated structure: (**a**) SSC particle scaffold on GDC pellet, (**b**) infusing GDC precursor solutions, (**c**) discrete GDC coating from GDC/glycine solution, and (**d**) continuous GDC coating from GDC/gelatin solution, after calcination at 800 °C.

Figure 1 describes the infiltration process and the resultant morphology of the coated GDC layers. The porous SSC structure absorbs the dropped solution, and becomes wet by capillary action. In order to enhance the surface adhesion of the metal precursor during the coating procedure, it is necessary to apply binder that can interact with the target inorganic material. From the previous researches on the utilizing of metal precursors for SOFCs, glycine was selected as a binder to compare with gelatin^20^. Glycine is a common amino acid that has been widely used as a dispersant and chelating agent for inorganic materials. Both organic materials have common active sites, such as amino and carboxyl group. Thus the ability to interact with ions and to form arranged complex will be the keys of film quality. After the coating process including infusion and sintering, the coated layers showed totally different structures. Figure 1c shows that the glycine-based coating resulted in sparsely distributed small nanoparticles, instead of uniformly covered film on the SSC nanoparticles. Since glycine is a simple amino acid with short molecule length, the complexes are not confined by surrounding materials, as they do not form networks. Hence, the glycine–GDC complex will independently accumulate on the surface to form a layer, and forms an agglomerated structure during drying and reduction, since a merged spherical shape is thermodynamically preferred. On the other hand, the infused precursor solution with gelatin formed a continuous morphology on the porous SSC cathode backbone, instead of agglomerated particles. The non-agglomerated surface can be related to the crystal growth of metal oxide between the confined complex layers, which was reported in previous research^9^. Since the metallic ions were captured by the side chains of gelatin molecule, such as amide, hydroxyl, and carboxyl groups, the metallic ions can be partially condensed to form membranes by salting out effect during the evaporation process^21^. Also, the metal ions are distributed uniformly by means of ionic interaction among amide groups on the gelatin molecule and metal ions, since the thermally stable property of gelatin facilitates the nanoscale thin film of GDC at the final stage of heating^10, 22, 23^.

**Figure 2:**
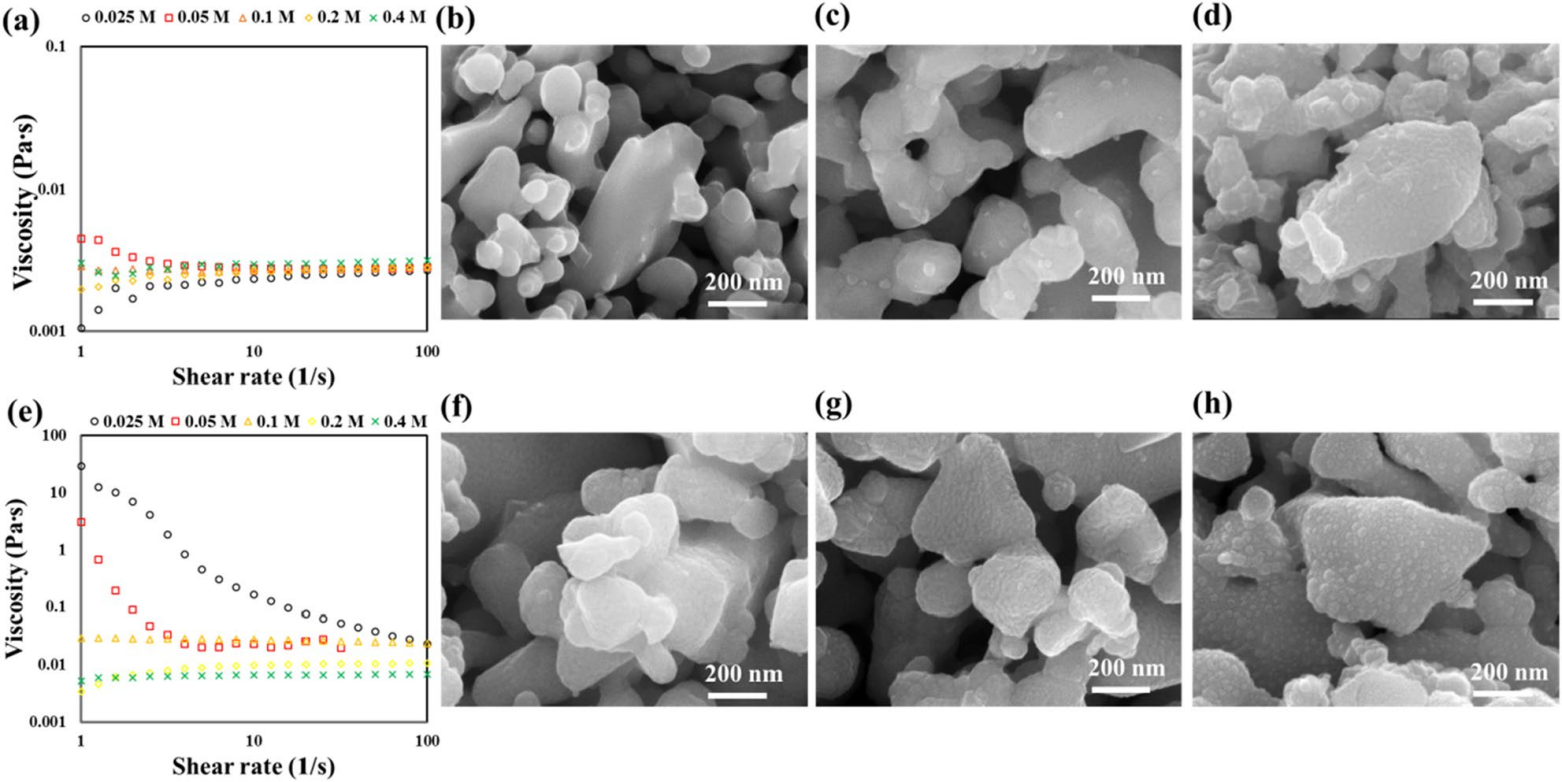
Viscosity of (**a**) glycine solution, and (**e**) gelatin solution, according to GDC precursor mole concentration. SEM images of the coating morphology from glycine/GDC (top) and gelatin/GDC (bottom) infiltration with the concentration of (**b**) and (**f**) 0.025 M, (**c**) and (**g**) 0.05 M, and (**d**) and (**h**) 0.4 M.

The change of viscosity with concentration of metal ion was compared to investigate the interaction between metal ion and organic additives. There are two considerations for the investigation of the rheology of ionic solution with organic additives. First, when a polar molecule is dissolved in a strong electrolyte, the Coulomb interaction (positively related to viscosity) between anion and cation weakens^24^. That means that the viscosity of ionic solution could be thinned by adding organic additives, such as amino acids and oligomers^25^. Secondly, the volume fraction of solute also affects the viscosity of solution, which in our case, is related to the concentration of organic additives^26^. The viscosity of glycine solution according to the GDC precursor molar concentration should be explained with consideration of those phenomena. In Fig. 2a, since the viscosity measurement at the low range of shear rate around 1 s^−1^ is usually unstable, the resultant viscosity was randomly achieved. The viscosity became stable over 5 s^−1^ of shear rate, which shows positive relationship with the GDC precursor molarity. As the volume fraction of glycine in the solution is negligible (< 0.005) and the viscosities are always higher than for pure solvent, the viscosity difference can be affected by the molar concentration of metal precursor and glycine–ion interaction. Since the solutions show Newtonian characteristic and the viscosity is almost linearly proportional to GDC molarity, the interference of Coulomb interaction by glycine was not a dominant factor for the viscosity adjustment. That means glycine does not sufficiently interact with the metal ions in the solution, which is related to the homogeneous distribution of the precursor ions. Since the metal precursor was not adequately caught by the dispersant, the coating from glycine-based solution shows non-uniform particulate distribution in the molar concentration range of 0.025–0.4 M as shown in Fig. 2b–d. At the lowest GDC concentration, the surface of backbone structure was not sufficiently covered, and bare SSC nanoparticle was observed. The amount of agglomerated GDC particles was increased with increasing the concentration, but it was not available to coat the surface uniformly.

On the other hand, the viscosity of gelatin solution according to the GDC precursor molar concentration shows a non-linear relationship. A mixture of gelatin and the solvent is naturally a gel state at RT, because of the crosslinking among gelatin triple helices. However, when an ionic precursor is added, the hydrogel will become flowable due to the metal ions being intercalated and coordinated at the organic ligands where the crosslinking was originated^27^. As shown in Fig. 2e, in the case of the (0.025 and 0.05) M solutions, the dynamic viscosity curves follow the behavior of non-Newtonian fluid^28, 29^. The shear thinning effects of those two solutions correspond to the behavior of the pseudoplastic material that can be found in typical hydrogels. Since the hydrogels have 3-dimentional network by hydrogen bonding, their mechanical properties are usually explained by viscoelasticity^30^. Therefore, the viscoelastic behavior of the solutions with low ionic concentration is the result of networks remaining inside the solution, and some portion of the active sites being occupied by the metal ions. The significant decrease of viscosity according to the increase of molarity means the metal precursor actively interacts with the gelatin molecules, and gets rid of the hydrogen bonding among gelatin molecules and solvent. Interestingly, with high molarity above 0.1 M, the solutions show Newtonian behavior, which is unusual for polymeric solutions. The Newtonian behavior implies that most of the gelatin molecules in triple helix in the solution are already denatured, due to the interaction with precursor ions, and some excessive ions will freely exist in the solution. In the SEM images of gelatin-assisted coating (Fig. 2f–h), the surface of SSC after being coated with lower GDC molarity shows uniform film morphology, while the highest molarity solution results in slightly aggregated layer. As previously mentioned, the gelatin solution showing non-Newtonian behavior with low GDC precursor concentration is partially denatured until the infusing process. After wetting the surface of SSC scaffold, the metal ion-containing gelatin forms a hydrogel–metal ion scaffold, due to the drying and condensation of solute^9, 10, 23^. The sufficient capacity of the active site of gelatin molecules and the capability of uniform distribution were the main reasons for the fine coating. In the case of higher GDC molarity, which has interconnection with Newtonian behavior, the metal ion–gelatin compound and excessive ions coexist in the solution; the resulting morphology was then continuous, but bulging, film.

**Figure 3:**
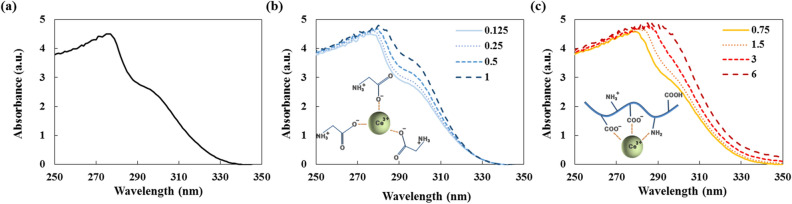
UV–Vis absorbance spectra of 0.05 M GDC precursor solution in (**a**) 60/40 aqueous ethanol, (**b**) glycine solution, and (**c**) gelatin solution. The legend indicates the wt% concentration of each organic molecule. The inset images describe the interacting elements in each solution.

UV–Vis spectroscopy for measuring the absorbance of metallic material was used to investigate the interaction between Ce^3+^ and the two organic additives^24–26^. The binding interaction could be monitored by the absorption spectrum, since the mechanism of UV–Vis spectroscopy is based on the electronic transition of absorbed radiation. In the measurement, the main factor for the change of absorbance peak is the concentration of the organic additives, due to the different amount of chelated cations. Therefore, once the molar concentration of metal ion is fixed, the degree of interaction between GDC cation and glycine or gelatin molecules can be compared in accordance with the concentration of the organic materials. It is known that the wavelength of the maximum of the Ce^3+^ absorption band is around 300 nm, which is consistent with our experimental result as shown in Fig. 3a^27^. It is expected that if the cations are sufficiently chelated, the shoulder peak will disappear^28, 29^. Though glycine is a popular chelating agent for its reactive carboxyl group, the absorbance peak of Ce^3+^ was not greatly affected by adding sufficient amount glycine according to the well-known glycine–nitrate method. The comparatively high concentrations of (0.5 and 1) wt%, which have higher mole fraction of reactive groups than metal ion, do not even suppress the absorbance peak as shown in Fig. 3b. On the other hand, the absorbance spectrum of Ce^3+^ in gelatin solution (Fig. 3c) shows significant suppression of the shoulder peak, which is in line with the result of previous investigation of the Fe^3+^–gelatin complex^10^. Since the mole fraction of reactive groups of 3 wt% gelatin solution is of a similar order to that of 0.5 wt% glycine solution, the chelating ability of gelatin on Ce^3+^ surpasses that of glycine. Consequently, we can conclude that the metal ions intercalated into the gelatin molecules weaken the hydrogen bonding by interacting with the amide groups. Due to the high coordinating capability of the functional groups, the interacting metal ions, such as Ce^4+^ and Gd^3+^, can be trapped along with gelatin molecules. Finally, during solvent evaporation and condensation, the cations are trapped between the interspace of gelatin membranes, and during thermal curing, grow to form dense and flat structure.

**Figure 4:**
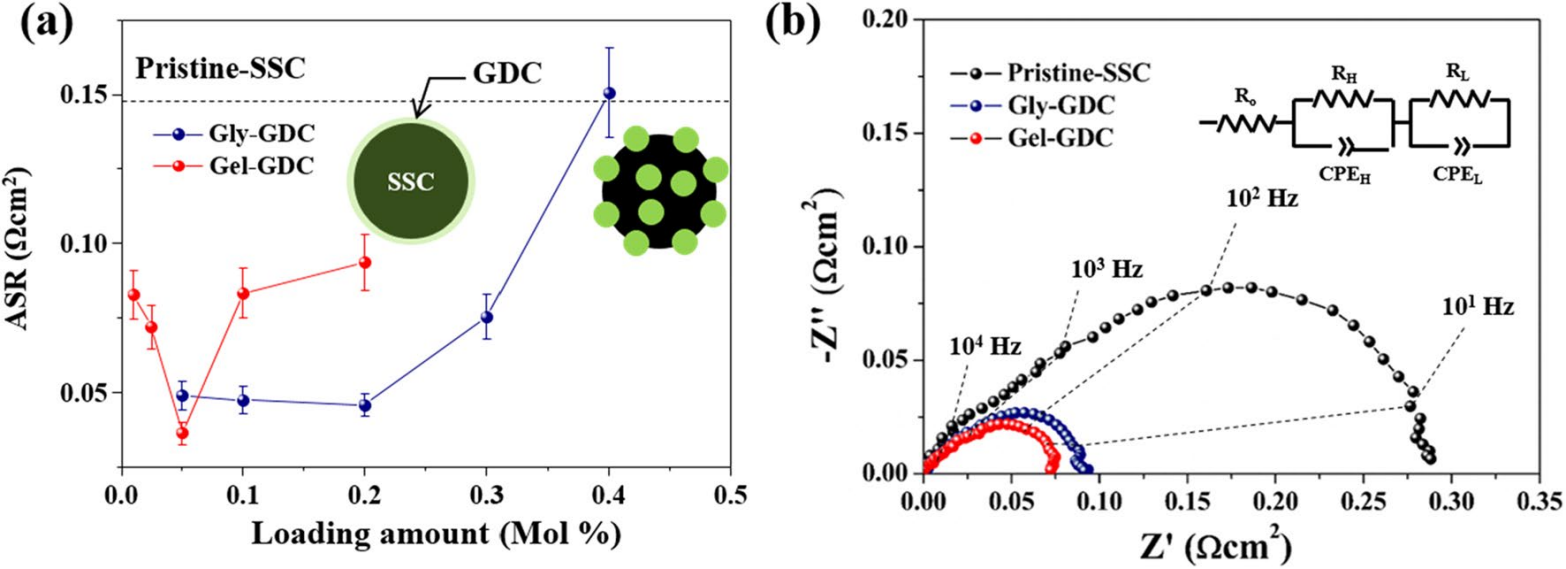
EIS results of Gly–GDC and Gel–GDC. (**a**) Rp of Gly–GDC and Gel–GDC as a function of the loading amount of GDC into the SSC cathode at 650 °C. (**b**) Representative impedance spectra of samples at optimized loading amount at 650 °C; Gly–GDC: 0.2 M, and Gel–GDC: 0.05 M.

Figure 4 shows the EIS results that were assessed in the symmetric cell configuration to compare the electrochemical behavior with respect to the two different morphologies of Gly–GDC and Gel–GDC. Figure 4a shows the variation of polarization resistances (R_p_), which is induced by resistance from electrochemical reactions in the electrode^31^, as a function of the loading amount of GDC into the SSC cathode. Regardless of the coated morphologies, R_p_ of the GDC-infiltrated SSC cathode is significantly reduced, compared to the Pristine-SSC. Interestingly, following the different morphologies of GDC, the trends of R_p_ depending on the loading amount are different. With GDC loading, R_p_s were substantially reduced up to optimum point, and it showed different points with respect to the morphologies of the GDC coating. In particular, the loading amount of GDC into the Gel–GDC was optimized with ~ 4-fold less amount, compared to that of Gly–GDC (Figure S5 of the Supplementary Information (SI) shows SEM images of Gly–GDC and Gel–GDC at the optimum loading amount). Furthermore, Fig. 4b, which show the Nyquist plot with equivalent circuit model (R-RC-RC), represents that R_p_ of Gel–GDC at that point is ~ 1.25 times smaller than that of Gly–GDC: R_p_,Gel–GDC: ~ 0.036 Ω cm^2^ and R_p_, Gly–GDC: ~ 0.045 Ω cm^2^ at 650 °C. Typically, discrete coating of ionic conductors, such as GDC here, demonstrated the substantial enhancement of the surface exchange reaction of cathode due to the enlargement of TPB^29, 30^. However, film coating of GDC layer onto the SSC cathode can induce the retarded surface reaction due to less exchange kinetics at the surface of GDC and blocked TPB with fully covered GDC layer. Nevertheless, several reports that coated the cathode material with fluorite material, such as ZrO_2_ and GDC layer in a thickness of < 5 nm, demonstrated the increased surface exchange reaction^14, 31^. Consequently, the results in Fig. 4 represent that the film coated GDC onto the SSC cathode (Gel–GDC) shows similar electrochemical performance to the Gly–GDC, without sacrificing the surface exchange of SSC, due to the full GDC coverage.

**Figure 5:**
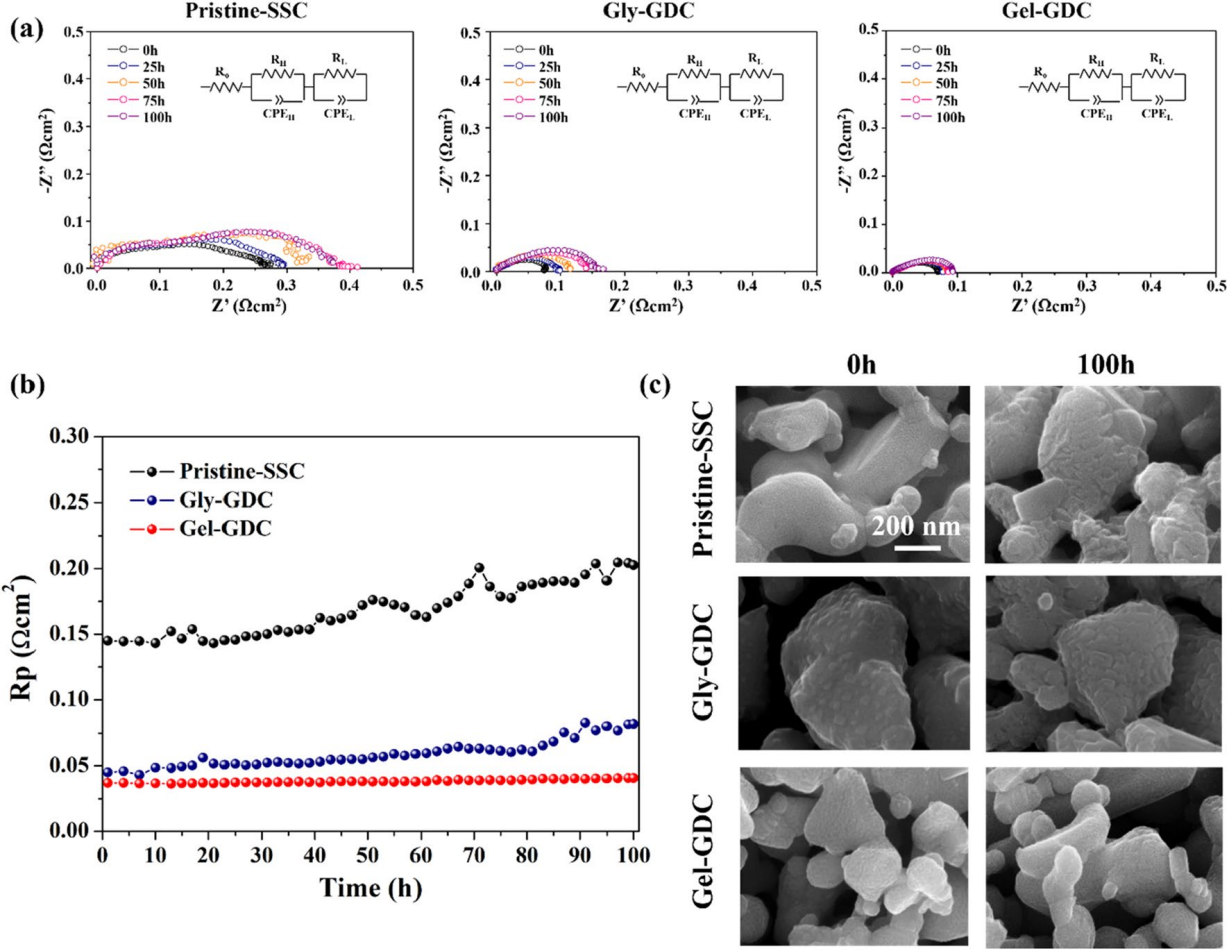
Stability test for 100 h at 650 °C. (**a**) Nyquist plots of Pristine-SSC, Gly–GDC, and Gel–GDC for long-term operation, (**b**) Variation of R_p_s for long-term operation, and (c) SEM images of the SSC with different coatings at 0 h and 100 h.

Moreover, to clarify the effect of GDC coating on the prevention of electrochemical stability, we assessed the stability test of the as-prepared samples of Pristine-SSC, Gly–GDC, and Gel–GDC for 100 h at 650 °C. Tests were performed with symmetric cell configuration in ambient air condition. Figure 5a represents the representative Nyquist plots with equivalent circuit model of each sample over 100 h. More quantitatively, Fig. 5b shows the R_p_ variations for 100 h of each sample. Under operation, the Pristine-SSC, Gly–GDC, and Gel–GDC demonstrate the R_p,f_/R_p,i_ of (~ 40, ~ 65, and < 10) %, respectively. Among them, Gel–GDC shows significantly enhanced electrochemical stability, compared with the Pristine-SSC and Gly–GDC. In terms of the performance degradation of the infiltrated electrode, the thermal agglomeration of infiltrated particles and cation segregation should be considered. However, since the thermal agglomeration of infiltrated particles at this temperature (650 °C) has been reported to not be a significant consideration, the major factor that governs the degradation is the cation segregation, especially Sr segregation, toward the surface. Figure 5c shows the morphological difference between the surface at (0 and 100) h of each sample. Gel–GDC represents no discernible changes in the surface morphology after 100 h, while other samples showed the morphological changes with generation of secondary clusters such as SrOx and Sr(OH)x (See Figure S6)^35–39^. Conformally coated fluorite layer can reduce the oxygen vacancy concentration at the perovskite surface, decreasing electrostatic interactions between charged defects, which attract Sr toward the surface^34, 39, 40^. Therefore, it can be ascribed that Gel–GDC reduce the oxygen vacancy concentration at the SSC cathode surface, resulting in suppressed Sr segregation with greatly enhanced electrochemical stability. On the other hands, Gly–GDC, which discretely covered the SSC cathode surface, did not effectively prevent the Sr segregation, resulting in the increased R_p_ and degraded cathode surface, as shown in Fig. 5b. We speculate that this is due to the open SSC surface without GDC coating, where the oxygen vacancy can still be enriched at the SSC surface, resulting in the Sr segregation^41^. Consequently, uniform and thin coating of GDC layer can greatly enhance the electrochemical stability, without sacrificing the electrochemical performance. In addition, it can decrease the amount of GDC loading to achieve the optimum performance, leading to cost effectiveness. As well as SOFC electrode, we believe that our new strategy with infiltration process assisted by gelatin can be further applied to other devices and structures that need to be coated using a wet chemical based technique.

## Conclusions

Biomolecules have received attention as functional materials for various applications, because Thin film GDC coating was attained in a thickness of only a few nanometers on the porous SSC cathode scaffolds by applying a hydrogel-based infiltration technique. The capability of interaction and coordination with metal ions were the key factors for coating quality. Compared with the conventional infiltration process based on monomer, especially glycine, the formation of well-defined film structure was available. With GDC/SSC hetero-interface, the performance enhancement can be achieved because of enlarged TPB on the cathode surface. Interestingly, as well as performance enhancement, it demonstrated the excellent stability at 650 °C for 100 h compared with non-GDC coated SSC cathode and discretely GDC coated SSC cathode with glycine. With surface analysis, the enhanced durability can be attributed to the suppression of Sr segregation by thin GDC layer.

## Supplementary Information

Supplementary information.
