# Supplementary material for: Infiltrated thin film structure with hydrogel-mediated precursor ink for durable SOFCs
Source: Sci Rep. 2021 Mar 29;11:7109. doi: 10.1038/s41598-021-86572-w (PMC8007576; doi:10.1038/s41598-021-86572-w)
Supplement: Supplementary file 1 — Supplementary information. [file 41598_2021_86572_MOESM1_ESM.docx]

Supporting material

Infiltrated thin film structure with hydrogel-mediated precursor ink for durable SOFCs

‡Sangyeon Hwang, ‡Mingi Choi, Giho Kang, Jongseo Lee, Seo Ju Kim, Baekhoon Seong, Hyungdong Lee, Wonyoung Lee*, and Doyoung Byun*

Departments of Mechanical Engineering, Sungkyunkwan University

‡These authors contributed equally.


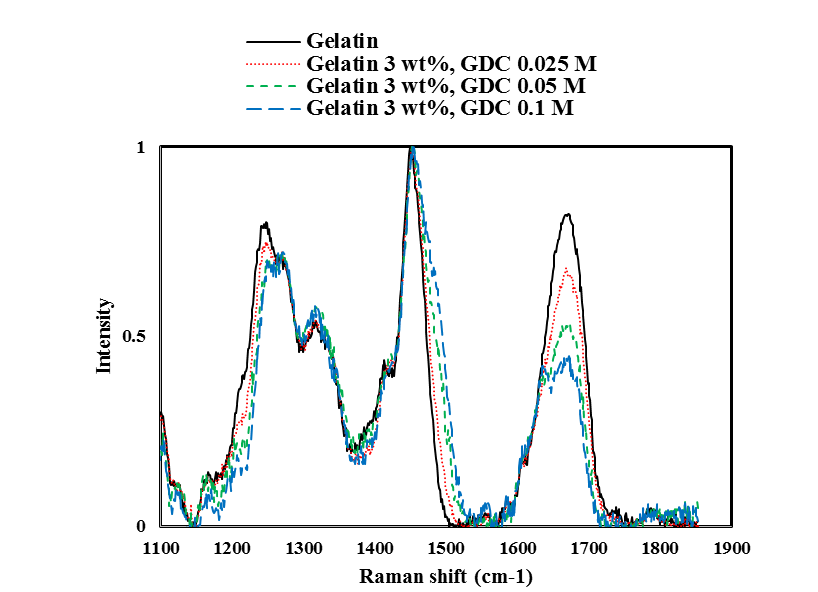


**Figure S1.** Raman spectrum of dried gelatin-ion compounds with 785 nm of wave length.

For the demonstration of the interaction, Raman spectrum of solutions with different precursor molar concentration was investigated. It was found that the peak related to the amide group (1650cm-1) decreases according to the addition of precursor concentration. This means the triple helix structure and secondary phase due to the intermolecular hydrogen bonding of the gelatin became extinct.


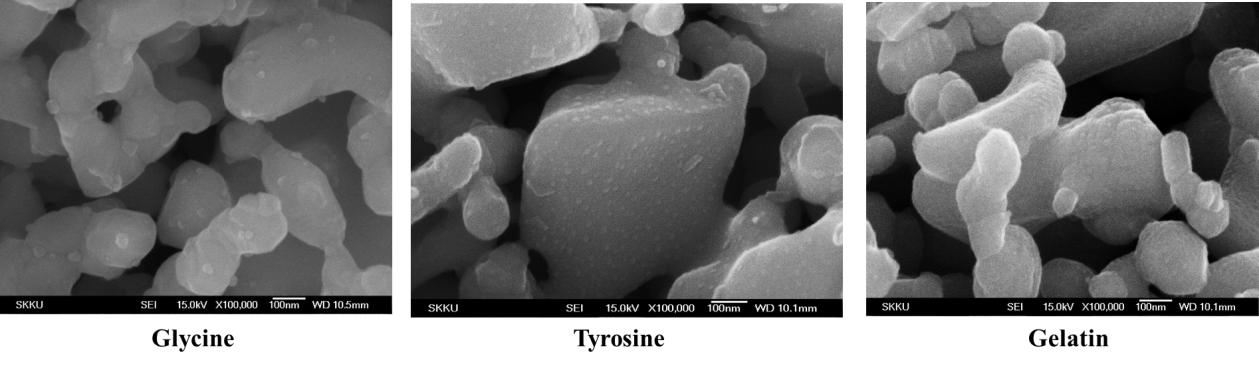


**Figure S2.** Morphology of GDC film coating from different materials.


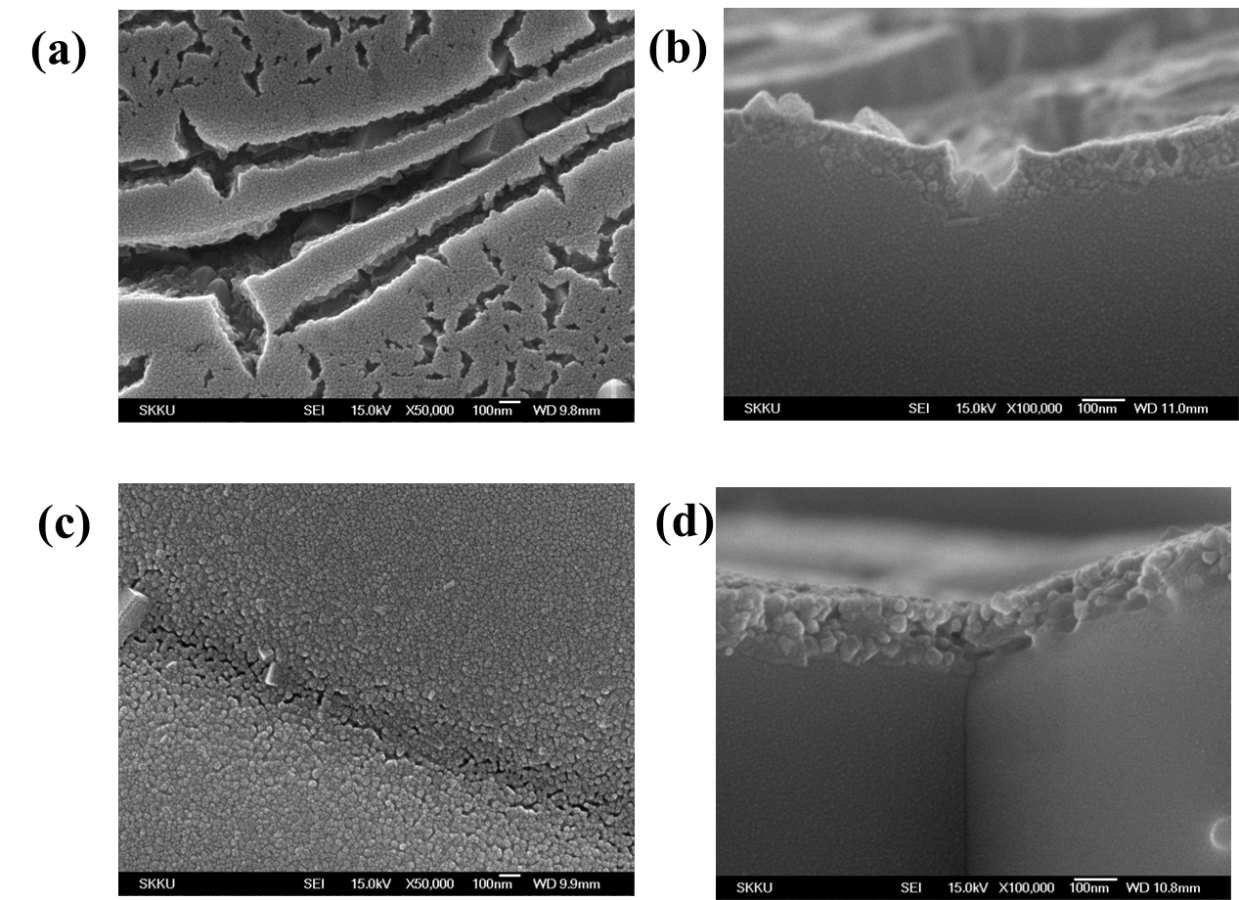


**Figure S3.** Comparison between glycine-based GDC layer and gelatin-based GDC layer with 0.4 M of GDC precursor. SEM images of the resultant film from (a) top view of glycine-GDC, (b) cross-sectional view of glycine-GDC, (c) top view of gelatin-GDC and (d) cross-sectional view of gelatin-GDC.


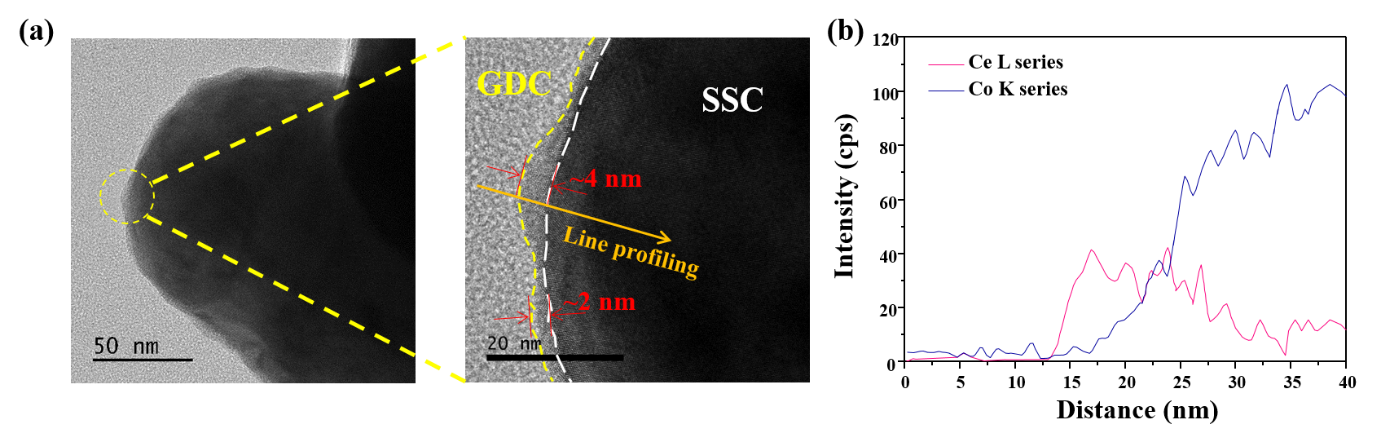


**Figure S4.** (a) TEM image of the SSC nanoparticle covered by gelatin-based GDC thin layer and (b) EDS line-profiling; Ce L and Co K series represent the GDC and SSC, respectively.


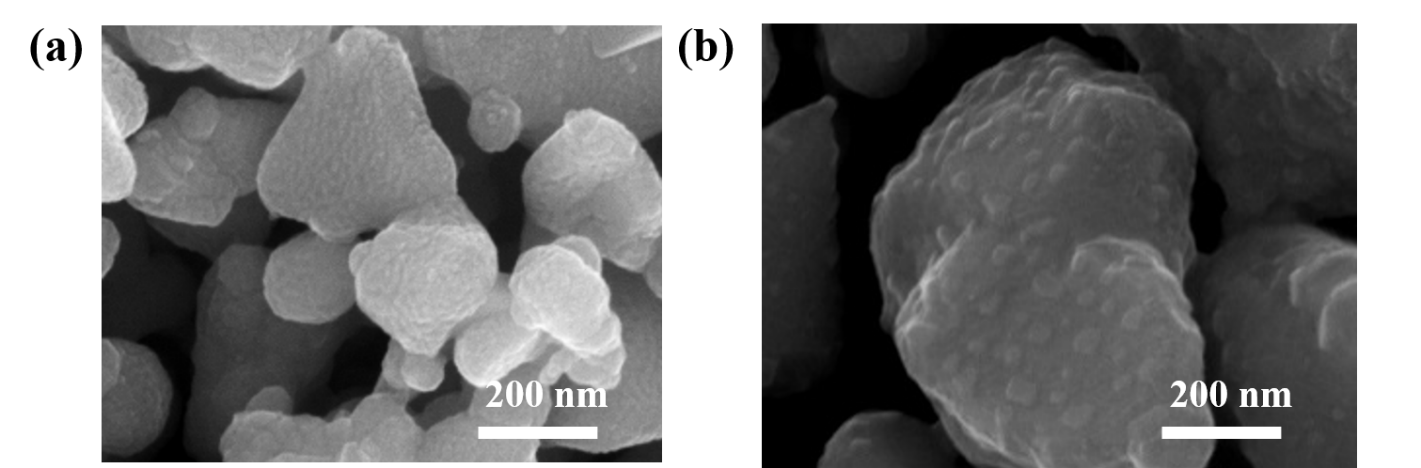


**Figure S5.** SEM images of (a) Gel-GDC (0.05 M) and (b) Gly-GDC (0.2 M) at the optimum loading amount.


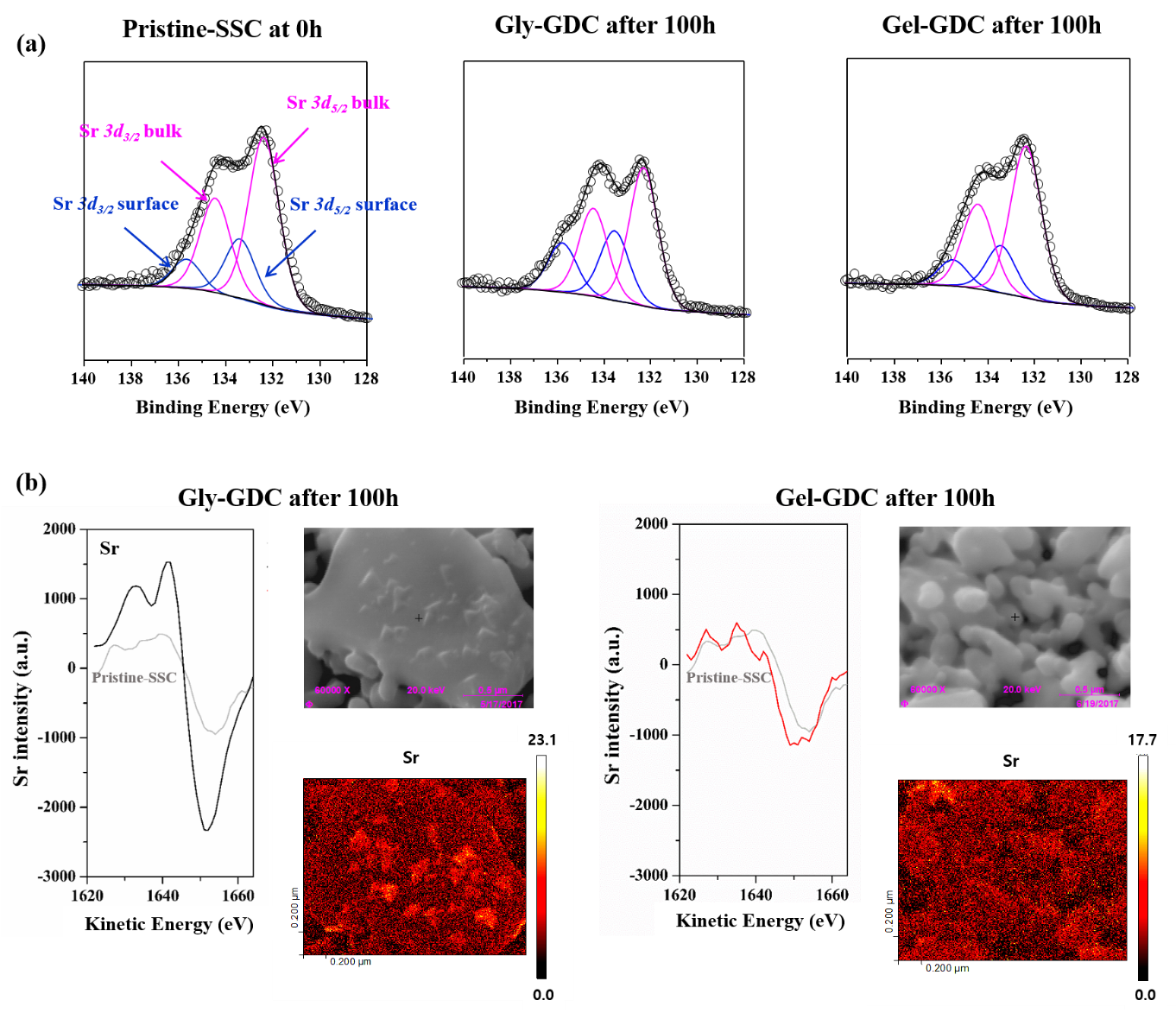


**Figure S6.** Characterization of Sr segregation at the surface after stability test. (a) Sr 3d photoelectron spectra in XPS, and (b) scanning of Sr *LMM* auger emission by AES.

To compare the degree of Sr segregation at the surface between Gly-GDC and Gel-GDC after stability test, Sr 3d photoelectron spectra in XPS and scanning of Sr *LMM* auger emission by Auger electron spectroscopy (AES) were characterized. Figure S6(a) represents the Sr 3d photoelectron spectra of Pristine-SSC at 0 h, Gly-GDC after 100 h, and Gel-GDC after 100 h. Sr 3d photoelectron spectra can be fitted into two sets involving the Sr 3d5/2 (lower energy set) and Sr 3d3/2 (higher energy set) with the energy separation of ~1.8 eV.^1-3^ Each energy set can be separated by Sr_surface_ and Sr_bulk_, and the ratio of Sr_surface_/Sr_bulk_ can be used as a quantitative measure of Sr segregation at the surface.^1,3^ Gly-GDC and Gel-GDC after 100 h showed the Sr_surface_/Sr_bulk_ values of ~0.848 and ~0.29, respectively, compared to that of ~0.32 at the surface of Pristine-SSC at 0h. Moreover, Figure S6(b) shows that the Sr *LMM* peak intensity measured by AES was higher in Gly-GDC after 100 h compared to that of Pristine-SSC at 0 h, while that in Gel-GDC after 100 h remained the similar. Unlike the substantial increase in Sr_surface_/Sr_bulk_ ratio and Sr *LMM* peak intensity in Gly-GDC after 100 h, the unchaged Sr_surface_/Sr_bulk_ ratio and Sr *LMM* peak intensity in Gel-GDC after 100 h further confirm the suppressed Sr segregation at the Gel-GDC surface.

**References**

1 Crumlin, E. J. *et al.* Surface strontium enrichment on highly active perovskites for oxygen electrocatalysis in solid oxide fuel cells. *Energy Environ. Sci.* **5**, 6081-6088 (2012).

2 Koo, B. *et al.* Enhanced oxygen exchange of perovskite oxide surfaces through strain-driven chemical stabilization. *Energy Environ. Sci.* **11**, 71-77 (2018).

3 Choi, M. *et al.* Engineering of Charged Defects at Perovskite Oxide Surfaces for Exceptionally Stable Solid Oxide Fuel Cell Electrodes. *ACS Appl. Mater. Interfaces* **12**, 21494-21504 (2020).
